# Analysis of VEGFR-2 and PDGFR-β expression in canine splenic hemangiosarcoma to identify drug repositioning candidates

**DOI:** 10.29374/2527-2179.bjvm001524

**Published:** 2024-08-06

**Authors:** Igor Simões Tiagua Vicente, Fernanda Barthelson Carvalho de Moura, Juliana Moreira Rozolen, Denner Santos dos Anjos, Renata Afonso Sobral, Carlos Eduardo Fonseca Alves

**Affiliations:** 1 Veterinarian, DSc., VetPrecision Laboratory, Botucatu, SP, Brazil; 2 Veterinarian, Departamento de Cirurgia Veterinária e Reprodução Animal, Escola de Medicina Veterinária e Zootecnia, Universidade Estadual de São Paulo (UNESP), Botucatu, SP, Brazil; 3 Veterinarian, MSc., Departamento de Cirurgia Veterinária e Reprodução Animal, Escola de Medicina Veterinária e Zootecnia, UNESP, Botucatu, SP, Brazil; 4 Veterinarian, DSc., Departamento de Cirurgia Veterinária e Reprodução Animal, Escola de Medicina Veterinária e Zootecnia, UNESP, Botucatu, SP, Brazil; 5 Veterinarian, DSc., Onco Cane Veterinary, São Paulo, SP, Brazil; 6 Veterinarian, DSc., Instituto de Oncologia Veterinária (IOVET), São Paulo, SP, Brazil

**Keywords:** dogs, spleen, endothelial cells, drug repurposing, cães, baço, células endoteliais, reposicionamento de fármacos

## Abstract

Splenic tumors are very common in dogs, and canine hemangiosarcoma (HSA) is one of the most important malignant splenic tumors. Surgery followed by chemotherapy (anthracycline-based protocols) is recommended for treating canine HSA; however, patients still do not achieve long-term survival. Therefore, this research aimed to assess vascular endothelial growth factor receptor-2 (*VEGFR-2*) and platelet-derived growth factor receptor-β (*PDGFR-β*) gene expression in formalin-fixed tissues, evaluate the quality of mRNA for quantitative polymerase chain reaction (qPCR) analysis and identify drug repositioning candidates based on VEGFR-2 and PDGFR-β. qPCR analysis identified the relative expression of heterogeneous VEGFR-2 and PDGFR-β, with samples showing no transcripts or very low expression and those with higher relative quantification for both genes. We then used immunohistochemistry to correlate the relative quantification of VEGFR-2 and PDGFR-β transcripts with respective higher protein expression to validate our results. In the next step, we evaluated drug repositioning candidates and identified small molecule inhibitors (i.e. sorafenib) and natural compounds (curcumin and resveratrol) with the ability to block VEGFR-2 and PDGFR-β genes. Overall, our results indicated that VEGFR-2 and PDGFR-β expression is highly variable among canine HSA samples and different drugs can block the expression of both genes. Therefore, a personalized approach could be useful for selecting anti-VEGFR-2 and PDGFR-β therapies and both genes are potential candidates for future oncological panels.

## Resumo

Os tumores esplênicos são muito comuns em cães, e o hemangiosarcoma (HSA) é um dos tumores esplênicos malignos mais importantes em cães. A cirurgia seguida de quimioterapia (protocolos baseados em antraciclinas) é a abordagem terapêutica mais recomendada para o tratamento da HSA canino; no entanto, os pacientes ainda não alcançam longa sobrevida após tratamento. Portanto, esta pesquisa teve como objetivo avaliar a expressão do receptor do fator de crescimento endotelial vascular-2 (VEGFR-2) e do receptor do fator de crescimento derivado de plaquetas-β (PDGFR-β) em tecidos fixados em formalina e identificar candidatos ao reposicionamento de medicamentos baseado na expressão desses genes. A análise qPCR identificou a expressão relativa heterogênea de VEGFR-2 e PDGFR-β, com amostras sem transcritos ou com expressão muito baixa ou amostras com alta quantificação relativa para ambos os genes. Em seguida, foi realizada o exame imuno-histoquímico para correlacionar a quantificação relativa dos transcritos de VEGFR-2 e PDGFR-β com a respectiva maior expressão proteica para validar nossos resultados. Na próxima etapa, avaliamos candidatos ao reposicionamento de medicamentos e identificamos inibidores de moléculas pequenas (ou seja, sorafenibe) e compostos naturais (curcumina e resveratrol) com capacidade de bloquear os genes VEGFR-2 e PDGFR-β. No geral, nossos resultados indicaram que a expressão de VEGFR-2 e PDGFR-β é altamente variável entre amostras caninas de HSA e diferentes drogas podem bloquear a expressão de ambos os genes. Portanto, uma abordagem personalizada poderia ser útil para selecionar terapias anti-VEGFR-2 e PDGFR-β e ambos os genes são potenciais candidatos para futuros painéis oncológicos.

## Introduction

Canine splenic hemangiosarcoma (HSA) is a malignant tumor originating from endothelial cells and exhibits aggressive biological behavior (Carnio et al., 2020; Johnson et al., 1989; Pinello et al., 2022; Robinson et al., 2020). Interestingly, canine HSA can be considered as a model for human angiosarcoma studies, because of similar pathological and clinical behavior (Heishma et al., 2023). Surgical removal associated with anthracycline-based chemotherapy has been proposed for treatment of canine HSA; however, the overall patient survival is usually < 5 months on average (Rozolen et al., 2021). Therefore, there is an urgent need to develop new therapeutic strategies for these patients. Studies have shown that proteins are expressed in blood cells and are associated with tumor behavior. Vascular endothelial growth factor receptor 2 (*VEGFR-2*) and platelet-derived growth factor-beta (*PDGFR-β*) stimulate angiogenesis by inducing endothelial cell proliferation and migration (Lin et al., 2020; Restucci et al., 2002).

Interestingly, VEGFR-2 and PDGFR-β expression was previously assessed in canine HSA, which demonstrated the overexpression of both markers in a group of samples, with a heterogeneous pattern (Adachi et al., 2016; Asa et al., 2012). However, no association with clinical data or drug repositioning has been reported thus far. VEGFR-2 and PDGFR-β receptors are targets of different tyrosine kinase inhibitor (TKI) drugs, including sorafenib, toceranib, imatinib, and rivoceranib (Kang et al., 2024; Verma et al., 2024). In addition to TKI, researchers have demonstrated an interest in identifying natural compounds that block VEGFR-2 expression (Verma et al., 2024). Interestingly, VEGFR-2 mutations have previously been described in human (Murali et al., 2015; Painter et al., 2020) and canine (Megquier et al., 2019) angiosarcomas, demonstrating the importance of this receptor in both species. In canine HSA, PDGFR-β mutation was also implied in tumorigenesis (Asa et al., 2012), and PDGFR-β blockers (imatinib and dasatinib) were effective in blocking PDGFR-β phosphorylation, leading to reduced cell viability (Dickerson et al., 2013). Thus, previous studies have demonstrated the predictive potential of both receptors for canine HSA, and drug repositioning strategies may benefit dogs.

Drug repositioning is a technique used in human medicine, in which the use of a drug indicated for the treatment of any disease is different from the initial indication (Jourdan et al., 2020). The advantage of this approach is the use of drugs that have already been approved for the treatment of different conditions and with well-established pharmacokinetics and pharmacodynamics. This technique has already been used for canine HSA, wherein propranolol (a drug related to the treatment of heart conditions) has been used clinically (Saha et al., 2021; Terauchi et al., 2023). A major challenge in drug repositioning is the identification of target candidates. Molecular docking is one of the most effective methods for identifying new drugs (Luo et al., 2016). Therefore, a target was selected and the drug target structure was evaluated to predict the interaction between both (Parisi et al., 2020). In this approach, the first challenge was to select a candidate gene for each tumor model. To the best of the author´s knowledge, no previous studies have investigated the potential of VEGFR-2 and PDGFR-β as drug repositioning candidates. Thus, this research aimed to investigate VEGFR-2 and PDGFR-β expression in canine splenic HSA with a focus on the identification of gene overexpression, and applied a drug repositioning approach to identify possible drugs that downregulate both genes.

## Materials and methods

### Study design

This study retrospectively reviewed the archives from the Institute of Veterinary Oncology – IOVET, VetPrecision Laboratory and Onco Cane Veterinary Clinic for identifying patients with splenic HSA and hematomas. Our study evaluated gene expression using paraffin blocks. Since mRNA can be highly degraded due to tissue fixation and other factors can also increase degradation, we applied some inclusion criteria to minimize misinterpretation of the results. Only samples from dogs were selected for histopathological analysis to confirm the diagnosis, and tissue fragments were fixed for up to 48 hours. Paraffin blocks should have sufficient tumor samples for mRNA extraction. Moreover, this sample group was previously assessed for Ki67 expression, and we excluded samples with no expression in the internal controls to avoid over-fixation.

For splenic hematomas, we selected cases in which we had at least 3 years of follow-up and the patient had no sign of systemic disease to ensure that the diagnosis was not related to HSA heterogeneity. A total of 50 HSA cases were identified; however, we could ensure tumor fixation for up to 48 hours in only 22 cases. Although we used samples from our archive and did not use live animals, this study was approved by the Ethics Committee on the Use of Animals in Research (protocol code: 0204/2018).

### Clinical information

The main focus of this project was not to associate VEGFR-2 and PDGFR-β expression with clinical and pathological factors. However, because our archive presents complete information, we opted to retrieve this information. For staging, patients underwent imaging examinations (abdominal ultrasound, three-view thoracic radiography, and echocardiography) to perform tumor staging. Clinical staging was performed according to tumor size (T), presence of lymph node involvement (N), and distant metastasis (M) based on a modification of the Owen (1980) scheme.

### Morphological analysis and mRNA extraction

To avoid areas of hemorrhage and necrosis, a new tissue section was obtained from the paraffin blocks and stained with hematoxylin and eosin (H&E). Using an optical microscope, we evaluated the stained slides and selected the areas with the highest number of neoplastic cells. Using a permanent pen, we drew the area to be assessed on top of the coverslip. Subsequently, we cut five tissue sections of 7 μM. We overlapped the unstained slide with the H&E-marked slide, and using a scalpel, we obtained areas enriched with tumor cells. mRNA extraction was performed using the RecoverAll™ total nucleic acid kit (Ambion Life Technologies, Carlsbad, MA, USA) according to the manufacturer's instructions, and mRNA concentration was obtained using a nano spectrophotometer (Nano Drop™, ND-8000, Thermo Scientific, Carlsbad, MA, USA). Minimal mRNA quality was evaluated according to previously published recommendations (Kashofer et al., 2013).

### VEGFR-2 and PDGFR-β gene expression

To assess VEGFR-2 and PDGFR-β gene expression, the cDNA was synthesized from the extracted mRNA at a final volume of 20 µL. Each reaction mixture contained 1 µg total RNA treated with DNAse I (Life Technologies, Rockville, MD, USA), 200 U SuperScript III reverse transcriptase (Life Technologies), 4 µL 5× SuperScript First-Strand, 10 mM dNTPs (Life Technologies), 1 µL Oligo-(dT) 18 (500 ng/µL) (Life Technologies), 1 µL random hexamers (100 ng/µL) (Life Technologies), and 1 µL 0.1 M dithiothreitol (DTT) (Life Technologies). Reverse transcription was performed for 60 min at 50 °C, and the enzyme was inactivated for 15 min at 70 °C. PCR was performed as previously described by our research group (Rozolen et al., 2021). Three endogenous genes (actin beta (ACTB), glyceraldehyde-3-phosphate dehydrogenase (GAPDH), and hypoxanthine phosphoribosyltransferase (HPRT)) with previously provided sequences were used ((Rozolen et al., 2021). The VEGFR-2 and PDGFR-β forward and reverse, respectively were: F: 5'GGCTACCAGTCCGGCTATCA 3' and R: 5'CCTCCTCGCTGGAGTACACAGT 3'; F: 5’ CCTCCCACATACGCTCCATT 3’ and R: 5’GTCCCTGAATCGCCAAGCT 3’. The reaction was conducted in a total volume of 10 µL containing Power SYBR Green PCR Master Mix (Applied Biosystems, Foster City, CA, USA), 1 µL cDNA (1:10), and 0.3 µM of each primer pair, in triplicate, using QuantStudio 12 K Flex Thermal Cycler equipment (Applied Biosystems). A dissociation curve was generated for each experiment to assess the specificity of PCR products. All experiments were conducted in triplicates. The mRNA extracted from 10 hematomas was used for normalization.

### Immunohistochemistry

VEGFR-2 and PDGFR-β immunoexpression was assessed only to ensure a positive relation between gene and protein expression. The selected antibodies were tested in canine HSA tissue following the previously described protocol (Adachi et al., 2016). Briefly, antigen retrieval was performed using a citrate buffer (pH 6.0) in a pressure cooker (Muscae Plus; Erviegas, Indaiatuba, Brazil). Endogenous peroxidase blockage was performed for VEGFR-2 and PDGFR-β antibodies using 8% hydrogen peroxide diluted in methyl alcohol for 1 h. The mouse monoclonal VEGFR-2 antibody (Clone A-3, Santa Cruz Biotechnology, Dallas, TX, USA) was used at 1:250 dilution and the rabbit polyclonal PDGFR-β (Rabbit polyclonal, Cell Signaling, Danvers MA; USA) at 1:1,000 dilution. IHC evaluation was performed as previously described (Adachi et al., 2016). Briefly, three random fields were evaluated and the percentage area of positive cells was scored as 0: < 10% of positively labeled cells; 1+: 10% to < 30% of positive cells; 2+: 30% to < 50% of positive cells; and 3+: ≥ 50% of positive cells.

### Drug repositioning approach

The Drug Gene Budger (DGB) was used (Wang et al., 2019) for identifying new drugs. Briefly, we assessed the online tool (Icahn School of Medicine at Mount Sinai, 2023a) and entered our target genes into a search tool. A list of drugs was generated, and we retrieved information from the Crowd Extracted Expression of Differential Signatures (CREEDS)-associated database. This analysis was focused on drugs that downregulate VEGFR-2 and PDGFR-β. We also used the L1000FWD online tool (Icahn School of Medicine at Mount Sinai, 2023b) to evaluate the interactive visualization of different small molecules regarding VEGFR-2 and PDGFR-β gene signature.

### Statistical analysis

First, we conducted a normalization test to ascertain the fundamental nature of our dataset, aiming to discern whether it adhered to parametric or non-parametric characteristics (Supplementary Table 1). The normalization curve classified the data as non-parametric. Then, for statistical purposes, we grouped the samples according to the clinicopathological criteria (age, breed, histological subtype, and survival) and compared different categorical variables with overall survival (OS). Categorical variables included histological subtype, metastatic status, clinical stage, and treatment. Spearman’s correlation was performed to evaluate the correlation among all variables, and a matrix of multiple correlations was constructed using the Spearman’s test. Survival analysis was performed using the log-rank test and Kaplan-Meier curves. Survival data were censored when the dogs were still alive or had died from other causes. Statistical analyses were performed using GraphPad Prism v8.1.0 (GraphPad Software Inc., La Jolla, CA, USA). Survival information was censored when the patients were still alive or had died of other causes. Statistical significance was set at p values < 0.05.

## Results

### Clinical information

Of the 22 patients, 12 were mixed-breed dogs, and 10 were purebred dogs. The mean age was 10.1 years (±1.7 years), and 72.7% (16/22) were female and 27.3% (6/22) were male dogs. Eleven patients had metastasis at diagnosis, while 12 dogs were not metastatic. The mean survival time was 114.2 days (±122.9 days). The most frequent histological subtype was cavernous (12/22), followed by solid (6/22), and capillary (4/22).

Regarding the therapeutic approach, two patients were suspected of having splenic HSA, but the owners did not accept surgery at first. These patients died from splenic rupture and were subjected to necropsy. Sixteen patients underwent surgery alone, and four patients underwent surgery plus chemotherapy with doxorubicin. The complete clinical information is shown in Supplementary Table 2.

### VEGFR-2 and PDGFR-β gene and protein expression

**Figure 1:**
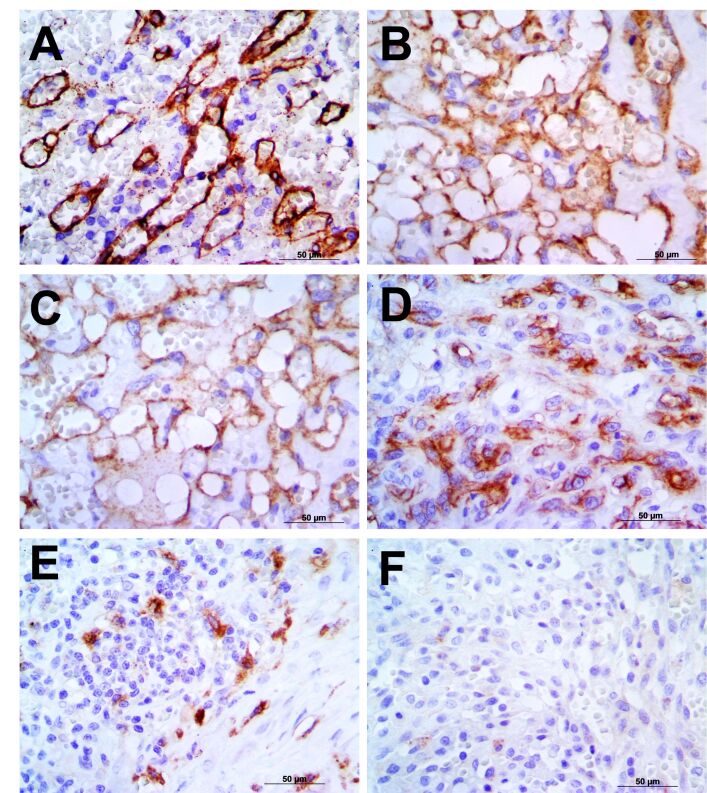
Representative immunohistochemistry scores for VEGFR-2 and PDGFR-β expression. (A) VEGFR-2 score of 4+ in a capillary histopathological subtype; (B) PDGFR-β expression in a capillary tumor with a score of 4+. Capillary HSA with a score of 3+ for VEGFR-2 (C) and PDGFR-β (D); (E) Solid HSA representing a score of 1+; (F) Solid HSA negative for PDGFR-β. Counterstain was performed with Harris Hematoxylin, 40x.

For this study, we selected three stable endogenous genes based on previous data from our research group. We then used ten hematomas to establish relative gene expression. *PDGFR-β* transcripts were detected in 18 out of 22 samples and *VEGFR-2* transcripts were detected in all 22 samples. The mean relative quantification of *PDGFR-*β was 11.67 (±16.9) and that of *VEGFR-2* was 10.2 (±41.8). Considering that gene expression is directly related to protein expression, we performed an immunohistochemistry (IHC) analysis and associated it with gene expression. Representative IHC scores for VEGFR-2 and PDGFR-β are shown in Figure 1. Samples with no detectable PDGFR-β transcript or very low transcript detection (< 0.05) did not show protein expression. Samples with relative gene expression between 0.058 and 0.536 presented 1+ PDGFR-β protein expression. Samples with > 14.0 relative expression had 3+ or 4+ protein expression. Samples with relative VEGFR-2 expression between 0.212 and 0.693 had 1+ or 2+ protein expression. Most samples with a relative expression between 1.028 and 2.32 had 3+ protein expression, and samples > 3.946 relative expression had a score of 4+ for VEGFR-2 protein expression. The individual values of the relative expression and IHC scores are shown in Supplementary Table 2.

**Figure 2:**
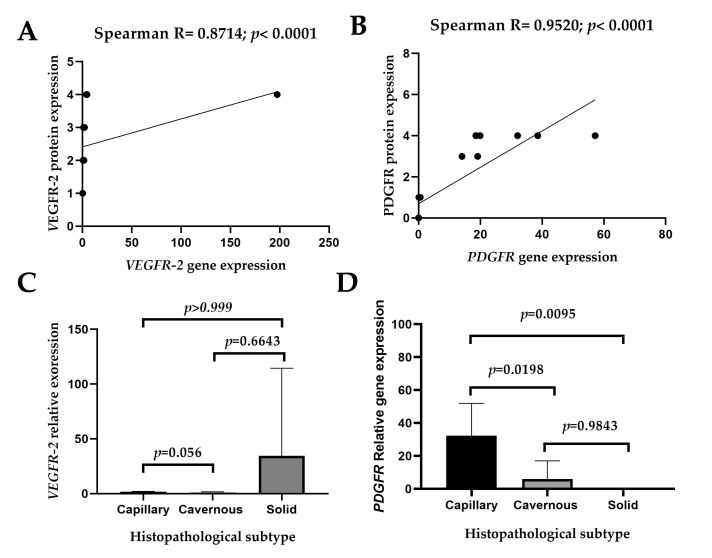
Statistical analysis of VEGFR-2 and PDGFR-β gene and protein expression in canine hemangiosarcoma (HSA). A positive correlation is observed between relative gene and protein expressions of VEGFR-2 (A) and PDGFR-β (B); (C) Absence of statistical difference when comparing VEGFR-2 transcripts with histopathological subtypes; (D) Strong association between histological subtypes and relative expression of PDGFR- β.

The primary focus of performing IHC analysis of each case was to ensure that the relative expression of VEGFR-2 and PDGFR-β was related to corresponding protein expression. We identified a strong correlation of VEGFR-2 (Figure 2A) and PDGFR- β (Figure 2B) gene and protein expression. Therefore, we confirmed that the relative expression of both the genes positively correlated with protein expression.

### VEGFR-2 and PDGFR-β gene expression and clinical factors

**Figure 3:**
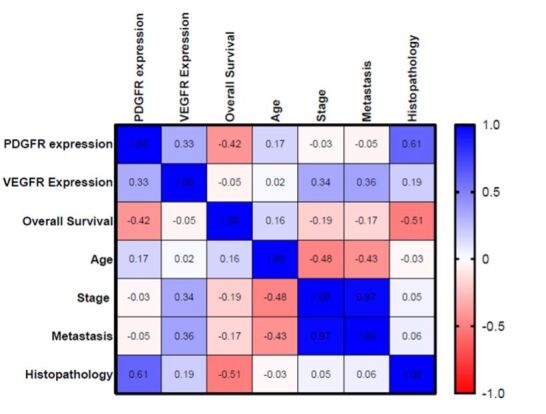
Multivariate analysis for VEGFR2 and PDGFR-β. When the expression of VEGFR2 and PDGFR-β receptors was assessed, there was a lower number of correlations with the patients´ clinical data. In the multivariate analysis, there was a moderate negative correlation between PDGFR-β expression and patient survival (R = -0.42). Younger patients showed moderate correlation with a higher probability of metastasis (R = -0.43).

Initially, our main focus was not to associate gene expression with clinical and pathological data. However, because this data was available, we opted to analyze this association. Univariate analysis showed no association of patients´ age, gender, tumor staging, and overall survival with the relative expression of VEGFR-2 and PDGFR-β. There was also no association between VEGFR-2 expression and histological subtype (Figure 2C). However, we identified a strong association between histopathological subtypes and PDGFR-β (Figure 2D). The capillary subtype showed the highest PDGFR-β transcript levels, followed by cavernous and solid with the lowest expression. Interestingly, this association was confirmed in the multivariate analysis, where capillary and cavernous subtypes showed a positive correlation with PDGFR-β relative expression. We also performed a multivariable matrix of correlation between each clinical factor and both VEGFR-2 and PDGFR-β expression. In this analysis, we identified that patients with the highest relative quantification of PDGFR-β had a shorter survival time (R = 0.42) (Figure 3).

**Figure 4:**
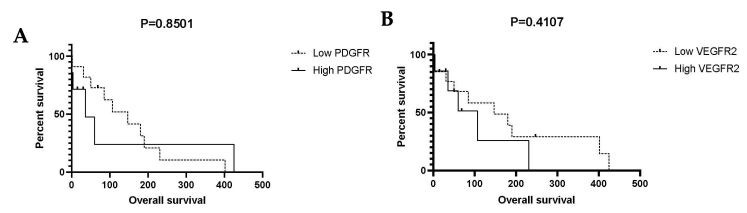
Survival analysis of VEGFR-2 and PDGFR-β gene expression and patient survival time. Expression of both the genes was not associated with patient survival.

Although our research did not focus on the association between gene expression and overall survival, since we identified a moderate correlation between PDGFR-β and survival, we segregated the samples into lower and higher survival; both VEGFR-2 (p = 0.4107) and PDGFR-β (p = 0.8501) did not show any association with patient survival (Figure 4).

### Identification of drug repositioning targets

We investigated VEGFR-2 and PDGFR-β gene and protein expression to identify where the expression of these genes could reflect in therapeutical implications. Based on the combination of gene and protein expression, we identified that samples with relative quantification > 1.0 showed protein overexpression (+3 or +4 expression for most of the samples). Our manuscript focuses on gene expression because we selected drug repositioning platforms that identify drugs modifying gene expression (up or downregulated gene expression). Because we selected two oncogenes, we focused our analysis on drugs that downregulated the expression of both genes. Based on expression profiles, the samples showed a heterogeneous expression pattern, indicating the need for a personalized approach for each patient. Based on association, we found seven samples overexpressing only VEGFR-2, two samples overexpressing only PDGFR-β, and five samples overexpressing both VEGFR-2 and PDGFR-β.

Using the drug repositioning approach for samples overexpressing VEGFR-2, we identified 636 drugs that could be used to downregulate this gene. Among these drugs, we found chemotherapeutics usually used for HSA, such as doxorubicin and vincristine; further, we found different small molecule inhibitors (including sorafenib and vemurafenib, which have previously established dosage in dogs) that are natural compounds with diverse clinical applications. Among drugs with other clinical applications, we highlighted isotretinoin (recently indicated for canine cutaneous lymphoma) and interferon (also indicated in some canine antitumor protocols). Among the natural compounds, we highlighted resveratrol and cucurbitacin-i because they have been studied in different cancer subtypes. A complete list of these compounds is provided in Supplementary Table 3.

For samples with PDGFR-β, we found 606 compounds that could downregulate its expression, and identified doxorubicin, vincristine, and chlorambucil as pharmacological options among chemotherapeutic agents. Sorafenib and vemurafenib were also identified as drugs that can downregulate PDGFR-β. Among drugs with other clinical use that can downregulate PDGFR-β, we highlighted digoxin, quetiapine, and sertraline as drugs with previously established dosages in dogs, and among the natural compounds curcumin and cucurbitacin-i were highlighted. The complete list of these compounds is provided in Supplementary Table 3.

**Figure 5:**
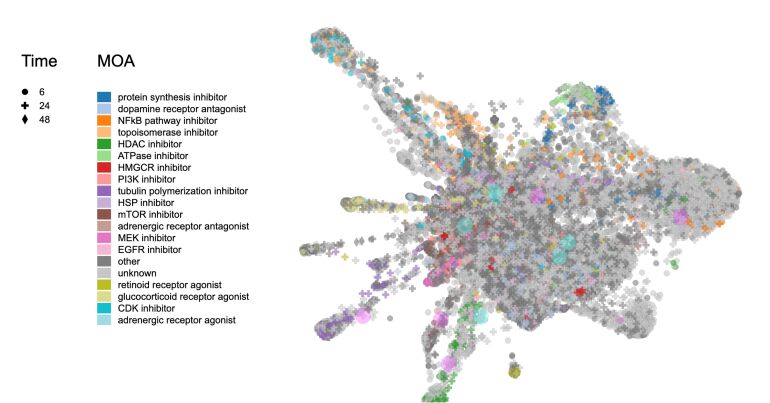
Representation of the different drug classes that can block VEGFR-2 and PDGFR-β expression.

We also assessed a gene signature considering the expression of both VEGFR-2 and PDGFR-β, focused on small molecule inhibitors (Figure 5). In this analysis, we identified several drug family agents that could block the expression of both genes, including phosphoinositide 3-kinase (PI3K) inhibitors, epidermal growth factor receptor (EGFR) inhibitors, mitogen-activated protein kinase kinase (MEK) inhibitors, and nuclear factor kappa B (NFkB) pathway inhibitors. Figure 1 summarizes the drug classes that can block this signature. We identified 16848 different molecules that could block both the genes. A complete list of drugs is shown in the Supplementary Table 4.

## Discussion

Our main research focus was to confirm that *VEGFR-2* and *PDGFR-β* gene expression is reliable, using paraffin samples and qPCR, and identify drug targets that could decrease the expression of both genes. *VEGFR-2* and *PDGFR-β* are genes related to tumor proliferation and we assessed gene expression of both to identify whether gene overexpression was associated with canine HSA. Our results identified a personalized expression, with patients showing *VEGFR-2* (N=7) or *PDGFR-β* (N=2) overexpression, patients overexpressing both genes (N=5), and patients with reduced expression of both genes (N=8). Since *VEGFR-2* and *PDGFR-β* expression can determine some therapeutic target drugs, our results indicate a personalized approach, with gene expression being assessed for each patient to indicate specific drugs for the patient’s treatment. Previously, the *VEGFR-2* and *PDGFR-β* gene expression was assessed by immunohistochemistry and the results also indicated a heterogenous pattern among samples (Adachi et al., 2016; Asa et al., 2012). However, no gene expression was assessed in these previous publications.

An interesting result was a group of samples with very high PDGFR-β (N=7) and VEGFR-2 (N=1) relative gene expression, with a value > 14 times that of hematomas. This result indicates that VEGFR-2 (N=7) or PDGFR-β tyrosine kinase inhibitors may be a therapeutical option. Our drug repositioning analysis identified linifanib, dabrafenib, vemurafenib, tivozanib, and erlotinib analogs, sorafenib, and selumetinib as small molecules capable of downregulating both genes. Vemurafenib (Rossman et al., 2021) and sorafenib (Cawley et al., 2022; Foskett et al., 2017; Marconato et al., 2020) have been tested in dogs with spontaneous cancer. Therefore, its pharmacokinetics, pharmacodynamics, and dosage for clinical use have been studied and are well established. Then, associating *VEGFR-2* and *PDGFR-β* gene and protein expression in canine HSA could increase its predictive value.

The manuscript approach is based on the use of IHC and qPCR using paraffin-embedded samples. In this type of ample, mRNA is usually highly degraded and molecular tests using this type of sample should be performed carefully. To avoid highly degraded material, we selected samples with fixation not > 48 h, and mRNA extraction and quality were performed according to the recommendations of a previous study (Kashofer et al., 2013).

To ensure a good qPCR investigation, we tested six endogenous primers in canine HSA samples and used the three most stable genes in this study. Endogenous genes are a very important step in qPCR analysis and we were very careful in this step to ensure analysis quality. The second important point relates to the sample used for the relative quantification of each transcript. The results of qPCR are a melting curve, and based on the time of amplification, it is possible to indicate how many times a sample has more transcripts for each related gene compared to the control (Livak & Schmittgen, 2001). Therefore, selecting a control for canine HSA can be challenging because no ideal normal sample is known. Because we are working with neoplastic tissue, the best control should be the corresponding normal (endothelial) cells, and obtaining the type of sample is not standardized for qPCR analysis. Splenic hematomas have been used as a control for qPCR assays involving canine splenic HAS. All criteria adopted in this study were made to ensure the minimal quality criteria and following the previous literature (Cheng et al., 2021; Rozolen et al., 2021; Tamburini et al., 2010).

We performed IHC analysis to evaluate protein expression and identify possible relationships between genes and protein expression. Our results demonstrate a strong correlation between gene and protein expression, indicating that higher transcript levels could also be correlated with higher protein levels. This step is important because proteins have a biological role in disease occurrence. The most important association of *VEGFR-2* and *PDGFR-β* was with histological subtypes. Subtypes with endothelial differentiation (capillary and cavernous) showed higher *VEGFR-2* and *PDGFR-β* gene expression. Moreover, the histological subtypes of HSA did not appear to be correlated with overall survival and other clinical characteristics.

VEGFR-2 and PDGFR-β are considered tyrosine kinase receptors and different inhibitors of both genes have been used in dogs with cancer. Sorafenib (Marconato et al., 2020), imatinib (Nakano et al., 2014), toceranib (Barnabe et al., 2013), and vermurafenib (Rossman et al., 2021) were previously tested in dogs, and their clinical efficacy, dosage, and toxicity have been well established. Toceranib has previously been tested in dogs with HSA and did not improve disease-free interval or overall survival in canine patients with stage I and II HSA (Gardner et al., 2015). However, the authors did not segregate patients based on VEGFR-2 and PDGFR-β. Because the expression of both genes is highly variable among patients, treatment based on gene expression profiles could increase the patients’ disease-free interval and overall survival.

Our drug target analysis also revealed some natural compounds that could block VEGFR-2 and/or PDGFR-β gene expression. From the author´s point of view, this is interesting because they are natural compounds with dosages previously established in dogs and could be associated with patients´ treatment. The use of natural compounds in dogs with cancer has grown globally among pet owners (Bianco et al., 2020). A very interesting research paper performed a global survey on the use of natural supplements in dogs with cancer compared to healthy dogs, including marine-derived omega-3 fatty acids, mushroom supplements, and curcumin (Bianco et al., 2020). As natural components can block both VEGFR-2 and PDGFR-β gene expression, we identified resveratrol and curcumin.

Resveratrol is a natural polyphenol that can be extracted from various plants. It is purported to possess anti-inflammatory, antioxidant, and cardioprotective properties, thereby reducing the risk of heart disease. In addition, resveratrol has been shown to exert anticancer effects (Ko et al., 2017). Interestingly, the antitumor activity of resveratrol has been assessed in canine HSA cell lines, alone or in combination with doxorubicin (Carlson et al., 2018). These authors demonstrated that resveratrol induces apoptosis in HSA cell lines and, interestingly, concluded that resveratrol increases the apoptotic effect of doxorubicin in canine HSA cell lines (Carlson et al., 2018). Therefore, resveratrol could be considered for the treatment of dogs with HSA. The safety, pharmacokinetics, pharmacodynamics, and dosage of resveratrol have been evaluated previously in dogs (Johnson et al., 2011). The initial test doses were 0, 200, 600, or 1200 mg resveratrol/kg/day for 90 days. The authors assessed the No Observed Adverse Effect Level (NOAEL) for resveratrol in dogs, and determined a dosage of 600 mg/kg/day. Therefore, clinical studies in dogs with cancer are necessary to establish an optimal dosage with in vivo antitumor activity.

Curcumin, a compound extracted from the rhizomes of Curcuma longa, has been reported to exhibit anti-inflammatory, metabolic, and neoplastic effects. In humans, it appears to help manage exercise-induced inflammation, muscle soreness, arthritis, anxiety, hyperlipidemia, and metabolic syndromes (Hewlings & Kalman, 2017). The antitumor effect of curcumin has been evaluated in canine cancer cells, and a previous study found that curcumin can induce apoptosis in canine mammary gland tumor cells (Turna et al., 2022). Interestingly, the pharmacokinetics, pharmacodynamics, toxic effects, and dosages of curcumin for periodontitis treatment have been previously demonstrated in dogs (Turna et al., 2022). Curcumin dosage in dogs ranges from 10 to 30 mg/kg (Campigotto et al., 2020; Deng et al., 2020). Since we identified curcumin as a potential blocker of VEGFR-2 and PDGFR-β, new clinical trials in dogs with cancer and overexpression of these genes are necessary to confirm this hypothesis.

In conclusion, the association of VEGFR-2 and PDGFR-β gene and protein expression can be predicted to the antitumor response of toceranib and other VEGFR-2 and PDGFR-β inhibitors.

## Supplementary Material

Supplementary material accompanies this paper.

Supplementary Table 1 The normality test demonstrated that our data do not fallow a Gaussian curve.

This material is available as part of the online article from https://doi.org/10.29374/2527-2179.bjvm001524
