# Supplementary material for: Analysis of VEGFR-2 and PDGFR-β expression in canine splenic hemangiosarcoma to identify drug repositioning candidates
Source: Braz J Vet Med. 2024 Aug 6;46:e001524. doi: 10.29374/2527-2179.bjvm001524 (PMC11315467; doi:10.29374/2527-2179.bjvm001524)
Supplement: Supplementary Table 1 [file bjvm-46-e001524-Suppl.pdf]

Supplementary Table 1. The normality test demonstrated that our data do not fallow a Gaussian curve.

**Test for normal distribution**	
Anderson-Darling test	
A2*	2,114
P value	<0,0001
Passed normality test (alpha=0.05)?	No
P value summary	****
D'Agostino & Pearson test	
K2	10,04
P value	0,0066
Passed normality test (alpha=0.05)?	No
P value summary	**
Shapiro-Wilk test	
W	0,7205
P value	0,0001
Passed normality test (alpha=0.05)?	No
P value summary	***
Kolmogorov-Smirnov test	
KS distance	0,3465
P value	<0,0001
Passed normality test (alpha=0.05)?	No
P value summary	****
Number of values	18
